# Supplementary material for: Pro-active monitoring and social interventions at community level mitigate the impact of coronavirus (COVID-19) epidemic on older adults’ mortality in Italy: A retrospective cohort analysis
Source: PLoS One. 2022 Jan 21;17(1):e0261523. doi: 10.1371/journal.pone.0261523 (PMC8782360; doi:10.1371/journal.pone.0261523)
Supplement: Attachment — Submitted filename: Answer to reviewers 31102021.docx [file pone.0261523.s003.docx]

Rome 31.10.2021

Dear Reviewers

please find below the answers to your suggestions that we included fully in the final manuscript. On behalf of the group of authors, I want to thank you for your contribution to improve the paper

with my best regards

Giuseppe Liotta

Reviewer #1: Thank you for your revision. While I agree to almost all author responses and revisions, there are still two minor comments that I think authors need to address and provide appropriate conceptual evidence before the manuscript qualifies for publication.

Thank you for your contribution to the improvement of the paper. Please find the answers to your comment by points

The authors have mentioned in response to comment 1 that mitigation and containment strategies by the government could have somewhat influenced the number of cases and mortality. Authors responded in the last sentence “Nevertheless, as suggested by the reviewer, we reported the latter consideration as a study limitation.”
Authors added the following sentence in the limitations part “adherence to governmental public health measures (wearing masks, keeping distance, stay at home, etc.; diseases affecting the studied population or the causes of death).” These measures were known to affect the infectiousness (Rt) overtime, thereby determining cases escalation or decrease over time, yet may cause the mortality rates to increase or decrease overtime if health systems were overwhelmed or cases were undetected. As authors agreed to the suggestion and noted in the limitation, it needs to be justified with evidence as conceptualized in the reviewer’s comments. The following evidence needs to be cited to in accordance to authors acceptance of the proposed work and need to be corroborated based on the above argument:
Ganasegeran, K.; Ch’ng, A.S.H.; Looi, I. What Is the Estimated COVID-19 Reproduction Number and the Proportion of the Population That Needs to Be Immunized to Achieve Herd Immunity in Malaysia? A Mathematical Epidemiology Synthesis. COVID 2021, 1, 13-19. https://doi.org/10.3390/covid1010003

Please see the discussion section, we have tried to report in the best possible way the considerations of the reviewer

Second, the SMR formula needs to be incorporated under methods part as an operational definition.

We added the SMR formula to the methods

Once these revisions have been made, I have no further objections for the paper to be published.

Reviewer #2: Thank you for your detailed replies to the reviewers. Supporting lonely isolated older people at risk is a key problem facing Western societies, Japan...

As has been stressed, you cannot make conclusions that the programme had an effect because of the large numbers of known and unknown confounders. I would like you to make a table listing all the confounders and items of data you were not able to collect listed by the two reviewers to illustrate to future researchers the data that they need to collect. This is not a criticism of your work, but important that as part of your scientific contribution your guide future researchers to identify methods to collect as much information on confounders as possible. Both reviewers listed large numbers of items of possible contacts that you were not able to assess

We added a table with the potential confounding factors w cannot assess at the end of the discussion section. thank you very much for your contribution
